# Midkine Deficiency Attenuates Lipopolysaccharide-Induced Pulmonary Inflammation

**DOI:** 10.3390/ijms26178519

**Published:** 2025-09-02

**Authors:** Yoshinori Tanino, Xintao Wang, Takefumi Nikaido, Yuki Sato, Ryuichi Togawa, Natsumi Watanabe, Mishie Tanino, Kenji Kadomatsu, Yoko Shibata

**Affiliations:** 1 Department of Pulmonary Medicine, Fukushima Medical University School of Medicine, Fukushima City 960-1295, Japan; xintaow@fmu.ac.jp (X.W.); taken@fmu.ac.jp (T.N.); yukisato@fmu.ac.jp (Y.S.); ryuichi@fmu.ac.jp (R.T.); natsumiw@fmu.ac.jp (N.W.); shibatay@fmu.ac.jp (Y.S.); 2 Department of Diagnostic Pathology, Asahikawa Medical University Hospital, Asahikawa 078-8510, Japan; mtanino@asahikawa-med.ac.jp; 3 Department of Biochemistry, Nagoya University School of Medicine, Nagoya 464-8601, Japan; kkadoma@med.nagoya-u.ac.jp; 4 Institute for Glyco-Core Research, Nagoya University, Nagoya 464-8601, Japan

**Keywords:** midkine, pulmonary inflammation, bronchial epithelial cells, chemokine, neutrophils

## Abstract

Midkine (MDK) is a multifunctional heparin-binding growth factor, and has been shown to regulate cell growth, survival, and migration. It also plays important roles in several inflammatory diseases such as sepsis. However, the role of MDK in the lungs has not yet been elucidated. In the present study, we investigated the role of MDK in pulmonary inflammation experiments using a mouse lipopolysaccharide (LPS)-induced pulmonary inflammation model and human bronchial cells. Wild-type and MDK-deficient mice were administered intratracheally with LPS, and several inflammatory parameters were analyzed. In the wild-type mice, MDK mRNA and protein in lung tissues were significantly increased after intratracheal LPS administration. The MDK-deficient mice showed significantly lower counts of total cells and neutrophils, as well as lower concentrations of total protein and neutrophil chemokines, KC and MIP-2 in bronchoalveolar lavage fluid, compared to wild-type mice. Moreover, mRNA expressions of TNF-α, keratinocyte chemoattractant (KC), and macrophage inflammatory protein (MIP)-2 in lung tissues, as well as the histopathological lung inflammation score, were significantly lower in the MDK-deficient mice. Furthermore, in in vitro experiments using bronchial epithelial cells, LPS stimulation increased mRNA expression of MDK, and MDK knockdown by siRNA decreased LPS-induced TNF-α and CXCL8 upregulation. These findings suggest that deficiency of MDK attenuates LPS-induced pulmonary inflammation, at least in part, through inhibiting inflammatory cytokine and chemokine upregulation in the lungs.

## 1. Introduction

Acute respiratory distress syndrome (ARDS) is a life-threatening respiratory failure triggered by several risk factors such as pneumonia and sepsis. ARDS is characterized by acute inflammatory lung injury with non-cardiogenic pulmonary edema [1,2]. There are no established pharmacologic therapies for ARDS, with reported mortality rates ranging from 35% to 46%. In the United States, approximately 190,000 patients are diagnosed annually, with a hospital mortality rate of 38.5% [3]. The prevalence of ARDS is reported to be 10% among patients in intensive care units and 23% among all ventilated patients [4]. The mechanism is complex, and various types of cells and mediators have been reported to be involved. There are several causes that induce ARDS, and the pathogenesis of ARDS may vary for each cause [5,6,7]. However, there are still many unclear points regarding the molecular pathophysiology of ARDS.

Midkine (MDK), discovered in 1988, is a low-molecular-weight heparin-binding protein with a molecular weight of about 13 coda [8]. It is highly expressed mainly in epithelial tissues in the process of epithelial-mesenchymal interactions, in differentiating nerve tissues, and in mesodermal tissues undergoing remodeling. Additionally, MDK plays roles in various physiological activities, including development, survival, and cell migration [8]. While MDK expression in adults is limited, it has been reported to be highly expressed in cancer cells [9,10,11] and the processes of inflammation and repair [12,13,14], as well as the pathology of various diseases [15,16,17,18].

There have been few reports showing the role of MDK in the lung. However, it has been reported that its expression is accelerated in the respiratory tract of cystic fibrosis patients [19], and that it has antimicrobial activity against bacteria and fungi [20,21,22]. While these findings suggest that MDK plays an important role in pulmonary inflammation, Zhang et al. have reported the role of MDK in ARDS-associated lung fibrosis [23]. However, the specific role of MDK in lung diseases remains unclear.

In the present study, to determine the role of MDK in pulmonary inflammation, we conducted in vivo experiments using a mouse lipopolysaccharide (LPS)-induced pulmonary inflammation model as well as in vitro experiments using bronchial cells which represent the first line of host defense against foreign inhaled components and play important roles in acute lung inflammation such as ARDS.

## 2. Results

### 2.1. Change in Midkine Expression in Lung Tissues After LPS Administration

**Figure 1:**
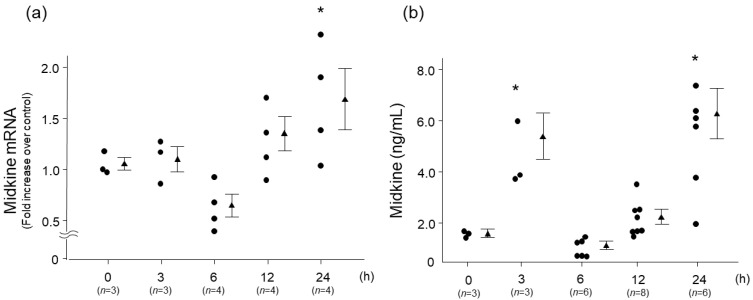
Midkine mRNA (**a**) and protein (**b**) expression in lung tissues after intratracheal lipopolysaccharide (LPS) administration into wild-type mice. In wild-type mice lung tissues, mRNA of midkine was significantly increased 24 h, and midkine protein was significantly increased 3 and 24 h after intratracheal LPS administration. Statistical differences between each group and 0 h were compared using ANOVA with Fisher’s least significant difference test as a post hoc test. * *p* < 0.05 vs. 0 h, circles: individual values, triangles: mean values of the indicated time points, mean ± SEM.

To characterize changes in the expression of midkine, wild-type (WT) mice were treated with LPS, and quantitative real-time PCR and ELISA were performed. Twenty-four hours after LPS treatment, the mRNA expression of MDK was significantly elevated (Figure 1a), and MDK protein in lung tissues was significantly elevated 3 and 24 h after LPS treatment compared to the baseline level (Figure 1b).

### 2.2. Bronchoalveolar Lavage Findings in Midkine-Deficient Mice

**Figure 2:**
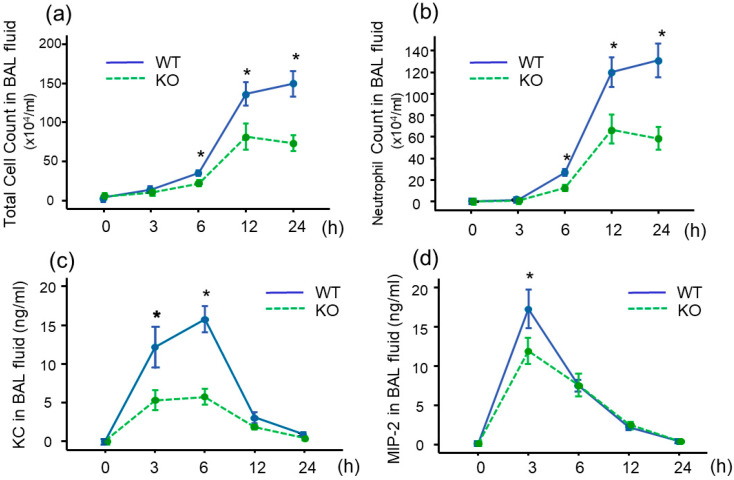
Bronchoalveolar lavage (BAL) fluid findings and chemokine levels after intratracheal LPS administration in midkine-deficient mice. Total cell (**a**) and neutrophil (**b**) counts in BAL fluid were significantly lower in midkine-deficient mice (KO) compared to wild-type mice (WT) 6, 12, and 24 h after LPS administration. The levels of keratinocyte chemoattractant (KC) (**c**) at 3 and 6 h and macrophage inflammatory protein (MIP)-2 (**d**) at 3 h were significantly lower in KO mice compared to WT mice. Statistical differences between WT and KO mice were compared using the Mann–Whitney U test. *n* = 5–16/group, * *p* < 0.05 vs. KO, mean ± SEM.

We administered LPS intratracheally into WT and MDK-deficient (*Mdk* KO) mice and analyzed bronchoalveolar lavage (BAL) fluid. At 6, 12, and 24 h after LPS administration, total cell (Figure 2a) and neutrophil (Figure 2b) counts in BAL fluid were significantly lower in the *Mdk* KO mice compared to the WT mice. Almost all the cells other than neutrophils in the BAL fluid were alveolar macrophages, and there was no difference in the number of alveolar macrophages between WT and *Mdk* KO mice. Few lymphocytes and eosinophils were observed.

### 2.3. Inflammatory Cytokine and Total Protein Concentrations in Bronchoalveolar Lavage Fluid of Midkine-Deficient Mice

**Figure 3:**
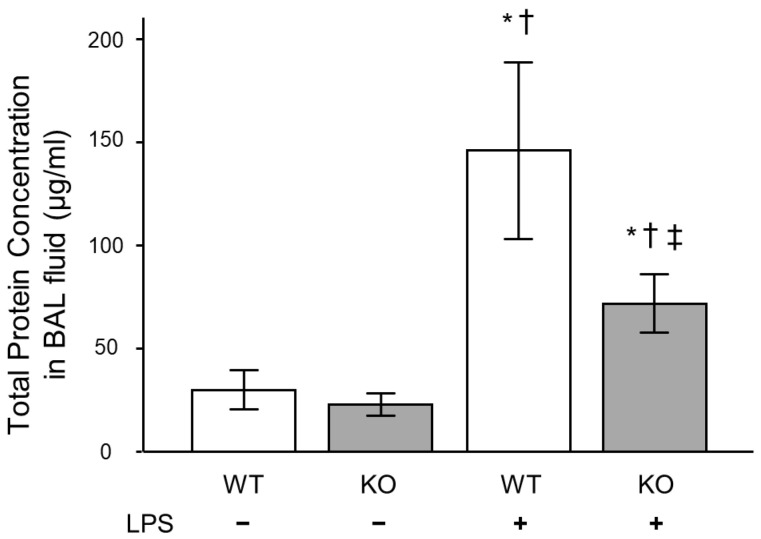
Total protein concentration in BAL fluid in midkine-deficient mice. Total protein concentration at 12 h was significantly lower in midkine-deficient mice (KO) compared to wild-type mice (WT). Total protein concentration in BAL fluid was compared using the ANOVA test and Fisher’s least significant difference test as a post hoc test. *n* = 5–8/group, * *p* < 0.05 vs. WT without LPS group, † *p* < 0.05 vs. KO without LPS group, ‡ *p* < 0.05 vs. WT with LPS group. Mean ± SEM.

To investigate the reason for decreased neutrophil lung inflammation in the *Mdk* KO mice, we analyzed the concentrations of keratinocyte chemoattractant (KC) and macrophage inflammatory protein (MIP)-2 in BAL fluid, which play critical roles in neutrophil recruitment into the lungs. The concentration of KC was significantly lower in the *Mdk* KO mice compared to the WT mice at 3 and 6 h after LPS treatment (Figure 2c). In addition, the concentration of MIP-2 was significantly lower in the *Mdk* KO mice compared to the WT mice at 3 h after LPS treatment (Figure 2d). Furthermore, total protein concentration in BAL fluid 12 h after LPS treatment was significantly lower in the *Mdk* KO mice (Figure 3).

### 2.4. Inflammatory Cytokine Expression in Lung Tissues of Midkine-Deficient Mice

**Figure 4:**
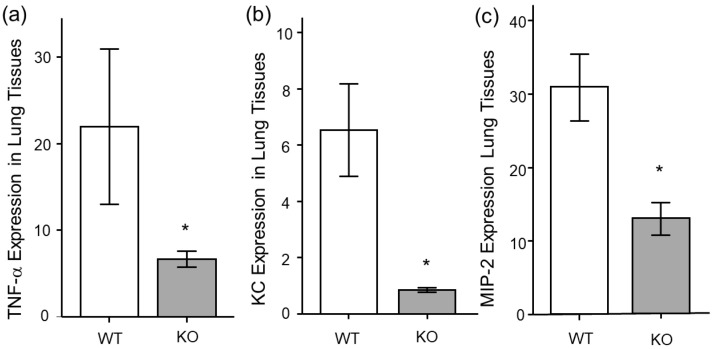
mRNA expression of inflammatory cytokines in lung tissues in midkine-deficient mice. mRNA expression of (**a**) TNF-α, (**b**) KC and (**c**) MIP-2 in lung tissues were lower in midkine-deficient mice (KO; *n* = 8) compared to wild-type mice (WT; *n* = 6) 3 h after LPS. mRNA expression was expressed as a fold increase over normal. Statistical differences between WT and KO mice were compared using the Student’s *t*-test. * *p* < 0.05 vs. WT. Mean ± SEM.

For the next analysis, we analyzed the mRNA expression of inflammatory cytokines in lung tissues. The mRNA expressions of TNF-α, KC, and MIP-2 were significantly lower in the *Mdk* KO mice compared to the WT mice at 3 h after LPS treatment (Figure 4a–c).

### 2.5. Histopathological Analysis of Midkine-Deficient Mice

**Figure 5:**
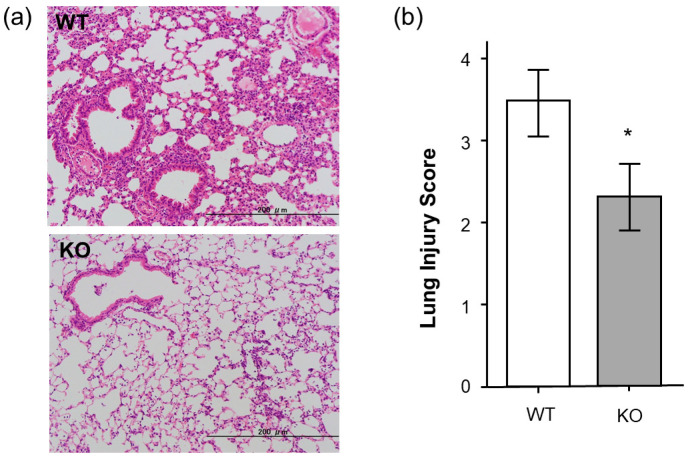
(**a**) Histopathological findings and (**b**) lung injury score in midkine-deficient mice. Lung inflammation was more severe in wild-type mice (WT) compared to midkine-deficient mice (KO) 24 h after LPS. Hematoxylin and eosin stain (**a**), and the lung injury score was significantly lower in KO mice compared to WT mice (2.3 ± 0.4 vs. 3.4 ± 0.4, bar: 200 µm, * *p* < 0.01; *n* = 6 for each group; mean ± SEM).

For further analysis, we compared the histological findings of lung tissues between the WT and *Mdk* KO mice. At 24 h after LPS administration, in the *Mdk* KO mice, pulmonary inflammation was decreased (Figure 5a), and the lung injury score was significantly lower compared to the WT mice (Figure 5b). These results suggest that LPS-induced pulmonary inflammation was suppressed in the *Mdk* KO mice.

### 2.6. Change in Midkine mRNA Expression in Bronchial Epithelial Cells After LPS Stimulation

**Figure 6:**
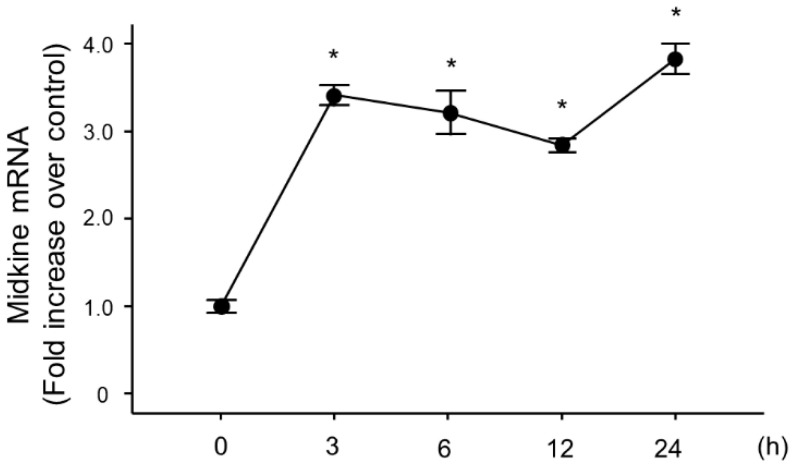
Midkine expression after LPS stimulation in bronchial epithelial cells. Midkine was significantly increased at and after 3 h post–LPS stimulation in BEAS-2B bronchial epithelial cells. Statistical differences between each time point and 0 h were compared using ANOVA with Fisher’s least significant difference test as a post hoc test. *n* = 4/group, * *p* < 0.05 vs. 0 h, mean ± SEM.

Because lung epithelial cells play important roles in pulmonary inflammation, we used BEAS-2B human bronchial cells to analyze the role of MDK in LPS-induced pulmonary inflammation. We stimulated the cells with LPS, and analyzed the changes in MDK mRNA expression, which revealed a significant increase at 3 h post-LPS stimulation (Figure 6).

### 2.7. Effect of Midkine Knockdown on LPS-Induced TNF-α and CXCL8 Expressions in Bronchial Epithelial Cells

**Figure 7:**
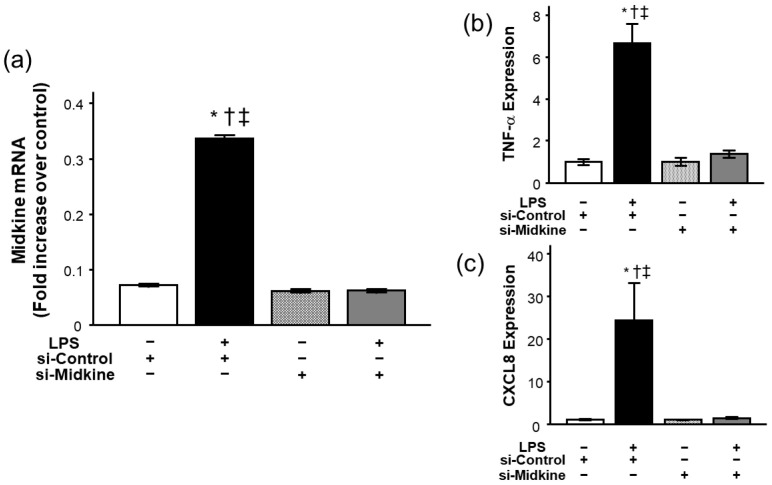
Effect of siRNA transfection on mRNA expression of midkine and effect of midkine knockdown on mRNA expression of inflammatory cytokines after LPS in bronchial epithelial cells. Transfection of midkine siRNA significantly inhibited upregulation of midkine mRNA 3 h after LPS (**a**). LPS significantly increased mRNA expression of TNF-α (**b**) and CXCL8 (**c**) in BEAS-2B cells. Knockdown of midkine by siRNA significantly inhibited upregulation of TNF-α and CXCL8 mRNA 3 h after LPS stimulation. mRNA expression of midkine and inflammatory cytokines in BEAS-2B bronchial epithelial cells was compared using the ANOVA test and Fisher’s least significant difference test as a post hoc test. *n* = 4/group, * *p* < 0.05 vs. si-Control group, † *p* < 0.05 vs. si-Midkine group, ‡ *p* < 0.05 vs. LPS plus si-Midkine group. Mean ± SEM.

For further evaluation of the role of MDK in pulmonary inflammation, we analyzed the effect of MDK knockdown on LPS-induced inflammatory cytokine expression in bronchial epithelial cells. We first confirmed that MDK mRNA expression was decreased to the baseline level 3 h after transfection of MDK small inhibitory RNA (siRNA) (Figure 7a). Among the groups, there was no difference in cell viability after siRNA treatment (>90%). Consistent with the results of *Mdk* KO mice, knockdown of MDK expression by MDK siRNA significantly decreased LPS-induced mRNA upregulation of TNF-α (Figure 7b) and CXCL8 (Figure 7c) 3 h after LPS stimulation.

## 3. Discussion

The present study demonstrated that MDK expression was increased in lung tissues after LPS administration in mice, and neutrophil accumulation in the lungs was significantly attenuated in *Mdk* KO mice compared with WT mice. In addition, expressions of inflammatory cytokines and chemokines, as well as the histopathological lung injury score, were significantly lower in *Mdk* KO mice compared with WT mice. Furthermore, in in vitro experiments using bronchial epithelial cells, MDK knockdown by siRNA decreased LPS-induced upregulation of TNF-α and CXCL8. These findings suggest that MDK exerts a pro-inflammatory effect in LPS-induced pulmonary inflammation.

MDK was first discovered as a heparin-binding cytokine that is highly expressed during embryogenesis [8]. Although MDK expression is relatively low in healthy adults, it has been found to increase during inflammation, tissue repair, and neoplastic transformation. Regarding inflammation, Sato et al. reported that MDK expression in renal proximal tubules was increased after renal ischemic reperfusion injury, and the numbers of neutrophils and macrophages infiltrating into the tubulointerstitium were significantly lower in *Mdk* KO mice than in WT mice [24]. Reduced renal damage with impaired infiltration of inflammatory cells into the renal tissue in the absence of MDK was also reported by other studies [25]. Moreover, in a mouse model of experimental autoimmune encephalomyelitis, infiltration of inflammatory cells into the spinal cord was decreased in MDK-deficient mice compared to WT mice [26]. These results indicate that MDK has a pro-inflammatory effect.

However, conflicting results have been reported as well. For example, Kojima et al. reported increases in inflammatory cell infiltration and matrix deposition in the glomerulus and the interstitium during the progression of crescentic glomerulonephritis induced by anti-glomerular basement membrane antibody in *Mdk* KO mice, showing that deficiency of MDK exacerbates necrotizing glomerular injuries in progressive glomerulonephritis [27]. It was also reported that knocking down endogenous MDK expression by siRNA enhanced TNF-α-induced apoptosis through activation of caspase-3 in prostatic cancer cells [28], and MDK had a protective role against cardiac ischemic/reperfusion injury [29]. Moreover, Takenaka et al. reported a protective effect of MDK in acute cardiac infarction [30], suggesting the possibility of organ-dependent effects of MDK.

In lung diseases, MDK is reported to be expressed in small airways, type II pneumocytes, and alveolar macrophages in COPD, as well as in the sputum of patients with ventilator-associated pneumonia caused by *S. aureus* [13]. Additionally, MDK expression has been demonstrated to be induced in the respiratory epithelium by hypoxia through hypoxia inducible factor-1α via a PKCγ-dependent pathway [31,32]. These findings suggest that MDK plays important roles in several lung diseases. In acute lung inflammation, plasma concentrations of MDK were found to be higher in patients with ARDS than in healthy volunteers [23], and were associated with pulmonary and kidney injury, as well as 28-day mortality in septic patients with ARDS [33]. An increase in serum MDK levels was also reported in patients with SARS-CoV-2 infection (COVID-19) [34].

To determine the role of MDK in acute lung inflammation, we used an LPS-induced pulmonary inflammation model. Our results showed an increase in lung inflammation in *Mdk* KO mice, consistent with previous studies. Xu et al. reported that MDK inhibition attenuated sepsis-induced lung injury via the angiotensin-converting enzyme/angiotensin II pathway, and demonstrated the role of MDK in pulmonary endothelial cells [35]. In the present study, we showed that neutrophil recruitment was attenuated with decreased levels of chemokines in the lung of *Mdk* KO mice when compared to WT mice. In addition, we focused on the role of MDK in bronchial epithelial cells, and demonstrated that LPS-induced upregulation of TNF-α and CXCL8 was attenuated by MDK knockdown in bronchial epithelial cells.

In the current study, we did not address several mechanistically relevant questions of interest. First, we analyzed epithelial cells because the cells play a critical role in pulmonary inflammation, and an increase in MDK had already been demonstrated in the cells [20,31]. However, we cannot exclude the possibility that other types of cells are involved in the pathogenesis of pulmonary inflammation, because MDK exists in a variety of cells. MDK expression has been shown in type II pneumocytes and alveolar macrophages in COPD [20], and increased expression of MDK protein due to hypoxia has been demonstrated in neutrophils, monocytes, and endothelial cells [36]. In the present study, MDK protein levels in lung tissues increased at 3 h, decreased subsequently, and showed a second peak at 24 h. Although the exact reasons for this biphasic pattern have not been clarified, it is possible that the difference in changes in MDK protein levels among the cells in the lungs is the cause of this discrepancy. Second, we showed attenuation of LPS-induced neutrophil chemokine upregulation in MDK inhibition. The low levels of chemokines in the lung are considered to be one of the mechanisms by which LPS-induced pulmonary inflammation was attenuated in *Mdk* KO mice. To the best of our knowledge, the present study is the first to report the role of MDK in the lungs, consistent with previous research investigating renal ischemia-reperfusion injury [25]. However, there are several other roles of MDK regarding neutrophil recruitment. MDK by itself was reported to act as a haptotactic and chemotactic agent for neutrophils [37]. In addition, MDK was shown to support neutrophil trafficking during acute inflammation by promoting adhesion via β2 integrin [38,39]. The relationship between MDK and neutrophil extracellular traps (NETosis) was also reported [40,41]. Furthermore, several mediators such as leukotriene B4 and complements were reported to be involved in the pathogenesis of acute lung injury [42,43,44,45]; we did not show the relationship between MDK and these mediators in the present study. Finally, we did not analyze the role of angiotensin-converting enzyme/angiotensin II pathway in the present study. Further study is required to fully elucidate the role of MDK in acute lung inflammation.

## 4. Materials and Methods

### 4.1. Reagents

The reagents used in this study were: *Escherichia coli* serotype 0111:B4 LPS (Signa-Aldrich, St. Louis, MO, USA), Power SYBR Green PCR Master Mix (Applied Biosystems, Foster City, CA, USA), BEAS-2B cells (ATCC, Manassas, VA, USA), mouse TNF-α DuoSet ELISA kit (R&D Systems, Minneapolis, MN, USA), mouse KC DuoSet ELISA kit (R&D Systems), mouse MIP-2 DuoSet ELISA kit (R&D Systems) and human IL-8/CXCL8 DuoSet ELISA kit (R&D Systems).

### 4.2. Animal Protocols

The Animal Research Committee of Fukushima Medical University approved all animal experiments (approved number: 25109, date: 19 November 2013). *Mdk* KO mice (obtained from Dr. K. Kadomatsu) used in this study had no gross abnormalities in the brain, lungs, heart, stomach, kidneys, testes, or ovaries by macroscopic and microscopic observation, consistent with Nakamura et al.’s study [46]. WT and *Mdk* KO mice were anesthetized with ketamine/xylazine, and 1 mg/kg of LPS was administered intratracheally. BAL was performed as previously described [47]. Briefly, a BD Insyte Autoguard catheter (Becton, Dickinson and Company, Franklin Lakes, NJ, USA) was inserted into the trachea, and 0.6 mL of physiological saline was infused a total of three times. After recovery of the fluid, the mice were sacrificed, and lung tissue was excised for use in analyzing the mRNA expression of mediators. In addition, for histopathological examinations, 10% formalin (Wako Pure Chemical Industries; Osaka, Japan) was administered into the trachea, and lung tissues were fixed with 25 cm H_2_O at 24 h after LPS administration.

### 4.3. Isolation of RNA

RNA was isolated with the Absolute RNA Miniprep Kit (Stratagene, La Jolla, CA, USA). Genomic DNA was digested with DNase I (Ambion, Austin, TX, USA), and RNA was reverse transcribed with the SuperScript III First-Strand Synthesis System (Invitrogen, Carlsbad, CA, USA).

### 4.4. Time Course of Midkine Expression

mRNA levels of MDK in lung tissues were analyzed by quantitative real-time PCR using the following primers: Fwd: 5′-CTCGCCCTTCTTGCCCTCTT-3′, Rev: 5′-GCAGGGCACCTTGCAATGGA-3′ as previously described [15].

### 4.5. Measurement of mRNA

Quantitative PCR was performed using Power SYBR Green PCR Master Mix and an ABI PRISM 7000 (Applied Biosystems). The threshold cycle (Ct) was calculated using Cts for the target genes and GAPDH. Relative mRNA expression was expressed as fold increase over values obtained from RNA from normal lungs or human reference total RNA (Stratagene) as previously described [48,49].

### 4.6. Preparation of Lung Homogenates

The whole lungs were homogenized and sonicated in 1.0 mL of the anti-proteinase-containing buffer (1 × PBS with 2 mM phenylmethylsulfonyl fluoride and 1 μg/mL each antipain, leupeptin, and pepstatin A), as previously described [47]. Specimens were centrifuged at 900× *g* for 15 min, filtered through a 0.45 μm pore-size sterile filter (Toyo Roshi, Tokyo, Japan), and frozen at −70 degrees until use for the measurement of the levels of cytokines in lung tissues.

### 4.7. Measurement of Midkine, Inflammatory Cytokine, and Protein Concentrations

The concentrations of midkine, human TNF-α, and CXCL8, as well as murine midkine, KC, and MIP-2, were measured with ELISA kits (Mouse Midkine ELISA kit, Abcam, Waltham, MA, USA, and DuoSet ELISA development kit, R&D Systems) according to the manufacturer’s protocols. Protein concentrations in mouse BAL fluids were determined using a BCA Protein Assay Kit (Thermo Fisher Scientific; Rockford, IL, USA).

### 4.8. Pathological Evaluation of Lung Sections

Pathological evaluation was performed as previously described [47]. Briefly, the lungs were excised and fixed by inflation at 25 cm of H_2_O with a phosphate buffer (10 mM, pH 7.4) containing 10% formalin for 24 h, and then embedded in paraffin. A 5 µm-thick tissue section was prepared and stained with hematoxylin and eosin. An observer who was blinded to the animal group assignment assessed 10 randomly chosen regions per tissue sample at a magnification of 400×, and scored lung damage severity as described previously [50]. Within each field, lung injury was scored for lung damage severity in each field based on two criteria: (a) neutrophil infiltration or aggregation in the airspace or vessel wall, and (b) alveolar wall thickness. Each criterion was evaluated on a 4-point scale (0 = no damage, 1 = mild damage, 2 = moderate damage, and 3 = severe damage). The sum of these scores was presented as the lung injury score.

### 4.9. Cell Culture

Human lung bronchial epithelial cells, BEAS-2B cells, were cultured in RPMI-1640 medium supplemented with 10% FBS (Gibco by Life Technologies, Grand Island, NY, USA), 100 IU/mL penicillin, and 100 μg/mL streptomycin (Sigma-Aldrich, St. Louis, MO, USA). After reaching 80% confluence, the cells were isolated, counted, and cultured in RPMI-1640 medium for 24 h. LPS (1.0 mg/mL) was added to the wells with or without knockdown of MDK, and the cells were incubated and harvested at specified time points.

### 4.10. Knockdown of Midkine

siRNAs were obtained from Thermo Scientific (ON-TARGET plus SMART human MDK and ON-TARGET plus Non-Targeting siRNA, Waltham, MA, USA). The nucleotide sequences of MDK siRNA were 5′-CGACUGCAAGUACAAGUUUUU-3′ and 5′-AAACUUGUACUUGCAGUCGUU-3′. Transfection of siRNAs was performed according to the manufacturer’s protocol as described previously [49]. BEAS-2B cells were incubated in growth medium for 24 h, and a final concentration of 100 nM siRNA was applied to the cells. Lipofectamine RNAiMAX (Invitrogen) was used as transfection medium. After 24 h, the cells were washed and incubated with or without LPS for 3 h.

### 4.11. Statistical Analysis

The Student’s *t*-test or the Mann–Whitney *U* test was used to compare two groups, while ANOVA was used to compare multiple groups. Fisher’s least significant difference test was used for post hoc analysis. For all analyses, *p* < 0.05 was considered statistically significant.

## 5. Conclusions

The results of the current study showed an increase in MDK in response to intratracheal LPS administration and reduced pulmonary inflammatory response in MDK-deficient mice following intratracheal LPS treatment. In addition, knockdown of MDK attenuated inflammatory responses in lung epithelial cells in vitro. Taken together, we conclude that deficiency of MDK attenuates acute lung inflammation, at least in part, through inhibiting inflammatory cytokine and chemokine upregulation in the lungs.

## Data Availability

The data presented in this study are available in the article.
